# Unilateral biportal endoscopy vs. open decompression for lumbar epidural lipomatosis-cohort study using a prospective registry

**DOI:** 10.3389/fneur.2024.1366357

**Published:** 2024-04-24

**Authors:** Bing Tan, Yu-hao Zheng, Chao Lei, Jian-yuan Ouyang, Yan-bo Wen, Zhuo-hua Shi, Qi-Yuan Yang

**Affiliations:** Department of Spine Surgery, The Third Hospital of Mianyang, Sichuan Mental Health Center, Mianyang, China

**Keywords:** lumbar epidural lipomatosis, lumbar spinal stenosis, UBE-ULBD, OLD, UBE

## Abstract

**Objective:**

This study aimed to compare the outcomes of unilateral biportal endoscopy, unilateral laminectomy bilateral decompression (UBE-ULBD), and open lumbar decompression (OLD) in patients with lumbar epidural lipomatosis (LEL).

**Methods:**

This prospective observational study was conducted from March 2019 to May 2022 and encompassed 33 patients with LEL who underwent lumbar decompression. The study included 15 cases of UBE-ULBD decompression and 18 cases of open decompression, which were followed up for 1 year. The baseline characteristics, initial clinical manifestations, and surgical details [including estimated blood loss (EBL) and preoperative complications] of all patients were recorded. Radiographic evaluation included the cross-sectional area (CSA) of the thecal sac and paraspinal muscles on MRI. Clinical results were analyzed using the Short Form-36 Score (SF-36), the Numeric Pain Rating Scale (NRS) for lumbar and leg pain, creatine kinase, the Roland and Morris Disability Questionnaire (RMDQ), and the Oswestry Disability Index (ODI).

**Results:**

The dural sac CSA increased considerably at the 1-year postoperative follow-up in both groups (*p* < 0.001). The operative duration in the OLD group (48.2 ± 7.2 min) was shorter than that in the UBE-ULBD group (67.7 ± 6.3 min, *p* < 0.001). The OLD group (97.2 ± 19.8 mL) was associated with more EBL than the UBE-ULBD group (40.6 ± 13.6 mL, *p* < 0.001). The duration of hospitalization in the OLD group (5.4 ± 1.3 days) was significantly longer compared with the UBE-ULBD group (3.5 ± 1.2 days, *p* < 0.01). The SF-36, NRS, RMDQ, and ODI scores improved in both groups postoperatively (*p* < 0.001). Serum creatine kinase values in the UBE-ULBD group (101.7 ± 15.5) were significantly lower than those in the OLD group (330.8 ± 28.1 U/L) 1 day after surgery (*p* < 0.001). The degree of paraspinal muscle atrophy in the UBE-ULBD group (4.81 ± 1.94) was significantly lower than that in the OLD group (12.15 ± 6.99) at 1 year (*p* < 0.001).

**Conclusion:**

UBE-ULBD and OLD demonstrated comparable clinical outcomes in treating LEL. However, UBE-ULBD surgery was associated with shorter hospital stays, lower rates of incision infection, lighter paravertebral muscle injury, and lower EBL than OLD surgery. Consequently, UBE-ULBD can be recommended in patients with LEL if conservative treatment fails.

## Introduction

The lumbar spinal canal fat filling the epidural space is a crucial component of the lumbar spinal canal that provides the dural sac with sufficient buffer space. However, excessive deposition of lumbar spinal canal fat compresses the lumbar dural sac and nerve root, causing nerve compression symptoms such as lumbar epidural lipomatosis (LEL) (1). The LEL can be idiopathic or secondary to Cushing's syndrome, obesity, or endocrine diseases, all of which are linked to excessive visceral fat accumulation (2). LEL is a rare disease in clinical practice; however, approximately 6% of symptomatic cases of lumbar spinal stenosis are associated with LEL (2, 3). Clinical manifestations include nerve root and cauda equina compression injury symptoms, such as neurogenic claudication, loss of sensation, lower limb weakness, cauda equina syndrome, and so on (3, 4). If symptoms are mild, conservative treatment should be administered. If the symptoms are severe combined with nerve or cauda equina compression symptoms, repeated conservative treatment is ineffective, or if the symptoms are progressively aggravated, surgical decompression is required (2, 3).

Recent studies have indicated that the diagnosis rate of LEF is only 8%, and it is easily missed or misdiagnosed in clinical practice, which may delay the optimal treatment time and affect a patient's quality of life (4, 5). Research on the diagnosis and treatment of LEF has increased in recent years, and numerous effective treatment methods have been proposed (6). It has been reported that 97% of the patients with LEF who undergo surgical treatment have improved symptoms (7). Currently, the surgical methods for LEF mainly include laminectomy, fat tissue removal, single-channel endoscopic-guided fat aspiration, spinal canal decompression, and intervertebral fusion decompression, all of which can achieve better clinical efficacy (8). A narrative review by Kim et al. (9) observed that most patients with SEL with acute symptoms who failed conservative treatment must undergo laminectomy and epidural fat removal to achieve good clinical efficacy. Bayerl et al. (10) have demonstrated that microscopic discectomy and decompression for LEF can result in favorable clinical outcomes. Traditional laminectomy and surgery to remove the excessive accumulation of adipose tissue requires wide incisions and extensive paravertebral muscle dissection to obtain a sufficient surgical field. However, this may lead to postoperative pain and a slow recovery (9, 10). Compared with open decompression surgery, minimally invasive endoscopic surgery is preferred by surgeons because of the shorter operation time, less intraoperative bleeding, fewer postoperative complications, and shorter postoperative recovery time (11). Studies have shown that single-channel spinal endoscopy exhibits optimal short-term clinical efficacy for treating LEF; however, it has a small operation space, narrow decompression range, difficult operation, and steep learning curve. These deficiencies limit the clinical application of single-channel endoscopy for LEF treatment (12, 13). Bilateral decompression is frequently required in patients with LEF complicated by severe lumbar spinal stenosis. However, the steep learning curve of single-channel spinal endoscopy and limited operating equipment consisting of a single rigid working cannula makes bilateral decompression difficult and high-risk (14). With the development of spinal endoscopic techniques, unilateral biportal endoscopy has been employed to treat severe lumbar spinal stenosis. Unilateral biportal endoscopy–unilateral laminectomy bilateral decompression (UBE-ULBD) is an effective, safe, and sufficient decompression surgery that can achieve excellent clinical efficacy for treating severe lumbar spinal stenosis (15). However, to date, no prospective study has compared the therapeutic effectiveness of UBE-ULBD and open lumbar decompression (OLD) for treating LEL, and the surgical approach remains controversial. This research aimed to investigate the clinical efficacy of UBE-ULBD for treating LEL and compare it with that of traditional OLD.

## Materials and methods

### Patient population

This study was approved by the Committee of Medical Ethics and Institutional Review Board of our hospital (Ethical 2023 Annual Review, No. 143). Patients were eligible if they fulfilled the following inclusion criteria: (1) conformed to the diagnostic criteria of LEL; had medical history and physical examination had corresponding symptoms of nerve root or cauda equina compression; the results of MRI examination disclosed that the epidural fat increased significantly, showing a continuous or shuttle band, with an anteroposterior diameter of more than 7 mm, and the dorsal dural sac was compressed, narrowed, or disappeared. All patients with LEL were graded according to the Ishikawa and Borré classification (6, 16). (2) The patient's conservative treatment for 3 months was ineffective, or the symptoms progressively aggravated. Patients were excluded for the following reasons: (1) lumbar spinal arteriovenous malformation, (2) intraspinal tumor, (3) death or loss to follow-up due to other diseases, and (4) history of lumbar surgery. A total of 33 patients were included in this study and were divided into a UBE-ULBD group and a traditional OLD group. All surgeries were performed by a single spinal surgeon. Demographic data, body mass index, follow-up time, surgical segment, operation time, complications, hospitalization time, and concomitant diseases were collected and compared between the two groups.

### Clinical and radiological assessment

Four clinical and radiological parameters were evaluated: (1) clinical results, (2) creatine kinase levels, (3) cross-sectional area (CSA) and paraspinal muscles, and (4) surgical complications.

Clinical results were analyzed using a questionnaire for outcome scores on the 11-point numerical rating scale (NRS) for leg and lumbar pain. The Oswestry Disability Index (ODI) and Roland and Morris Disability Questionnaire (RMDQ) scores were calculated preoperatively and at 1 week and 1 year postoperatively. Health-related quality of life was assessed using the Short Form-36 (SF-36) Health Survey. Odom's criteria were used to assess patient satisfaction. Creatine kinase levels were recorded before and at one and seven days after the operation.

Radiographic evaluation included the CSA of the thecal sac and paraspinal muscles based on MRI scans performed preoperatively and at 1 year of follow-up. The CSA of the thecal sac was measured at the disc level using T2-weighted axial MRI preoperatively and at 1-year follow-up. To eliminate inter-individual heterogeneity, the improvement rate of the dural sac CSA was analyzed. The improvement rate of dural sac CSA was calculated using the following formula: (postoperative dural sac area–preoperative dural sac area)/preoperative dural sac area × 100%. The multifidus and erector spinae muscles were measured, including the non-muscular tissue between them, together as one muscle unit, and considered the paraspinal muscles. The CSA of the paraspinal muscles was measured at the disc level using MRI preoperatively and at the 1-year follow-up using ImageJ software (NIH, Bethesda, MD, USA). The ratio of muscle CSA variation (RCV) was calculated according to the following formula: Last CSA/preoperative CSA × 100%. The degree of paravertebral muscle atrophy was calculated using the following formula: 100%–RCV.

### Surgical techniques

#### UBE-ULBD

**Figure 1:**
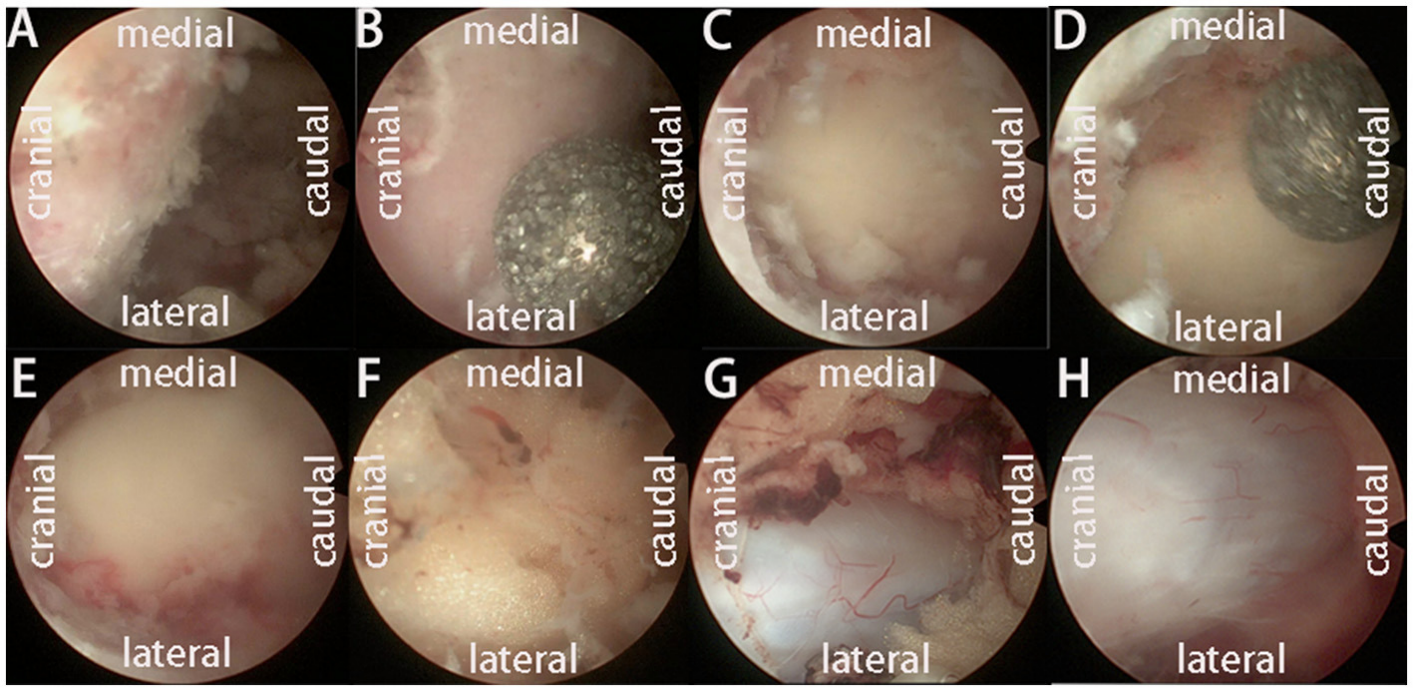
The operation process of UBE-ULBD technique: **(A)** Plasma cutter separates soft tissue on the surface of bony lamina and ligamentum flavum to establish an endoscopic workspace. **(B–E)** Using pliers bite, or grinding drill to remove the upper and lower edge of the bilateral lamina bone, exposing the starting and ending points of the ligamentum flavum. **(F, G)** The gun pliers completely bite off the ligamentum flavum and expose the abnormal hyperplasia of adipose tissue in the spinal canal. **(H)** After bilateral full decompression, the dura mater and nerve roots were released.

After general anesthesia, the prone position was taken, the target intervertebral space was determined and marked by C-arm fluoroscopy, the towels were routinely disinfected, the working channel was placed, and the segment and position were determined by fluoroscopy again. The bony boundary of the upper and lower laminae and the lateral facet joints were revealed. Decompression of the spinal canal was performed under a working channel. Hypertrophic ligamentum flavum and abnormal hyperplasia of adipose tissue were removed, and the dural edge and ipsilateral walking nerve root were exposed. Then, the contralateral ligamentum flavum and adipose tissue were removed, the contralateral nerve root was explored, and the bilateral dural sac edge and bilateral nerve root exploration were carefully examined to ensure no active bleeding (Figure 1). After the working channel was removed, a drainage tube was placed and sutured.

#### OLD

After general anesthesia, the prone position was taken, the target intervertebral space was determined and marked by C-arm fluoroscopy, the towels were routinely disinfected, and the segment and position were determined by fluoroscopy again. The bony boundary of the upper and lower laminae and the lateral facet joints were revealed. Decompression of the spinal canal was performed. Hypertrophic ligamentum flavum and abnormal hyperplasia of adipose tissue were removed, and the dural edge and ipsilateral walking nerve root were exposed. Then, the contralateral ligamentum flavum and adipose tissue were removed, the contralateral nerve root was explored, and the bilateral dural sac edge and bilateral nerve root exploration were carefully examined to ensure no active bleeding, a drainage tube was placed and sutured.

### Statistical analysis

All data are expressed as mean ± standard deviation unless otherwise specified. A board-certified spine surgeon blinded to the procedure evaluated all radiographic results. Interobserver reliability was assessed using intraclass correlations with data measured by one of the co-authors and classified as poor (0–0.39), moderate (0.40–0.74), or excellent (0.75–1.00). For continuous variables, within-group and between-group differences were detected using Student's and paired *t*-tests, respectively. Chi-square analysis was performed to compare categorical variables. Statistical significance was set at *P* < 0.05. All statistical analyses were performed using the SPSS software (version 23.0; SPSS Inc., Chicago, IL, USA).

## Results

### Demographic results

A total of 33 patients were diagnosed with LEL between March 2019 and May 2022, of which 15 were included in the UBE-ULBD group, and 18 observed at the same follow-up time interval were included in the OLD group. A summary of participants' demographic and baseline characteristics is presented in Table 1. The baseline demographic analysis (Table 1) showed no statistical differences between the two groups. Moreover, no differences were observed in the number of surgical segments or in perioperative complications. All patients with LEL were graded as grade 2 or 3 according to the Ishikawa classification and grade 2 or 3 according to the Borré classification. The OLD group had a shorter operative duration than the UBE-ULBD group (48.2 ± 7.2 min vs. 67.7 ± 6.3 min) and more estimated blood loss (EBL) (97.2 ± 19.8 mL vs. 40.6 ± 13.6 mL) in the UBE-ULBD group (*p* < 0.001, Table 2). The duration of hospitalization in the OLD group (5.4 ± 1.3 days) was significantly longer compared with the UBE-ULBD group (3.5 ± 1.2 days, *p* < 0.01, Table 2).

**Table 1:** Patients' demographic data.

**Variables**	**UBE-ULBD (*n* = 15)**	**OLD (*n* = 18)**	***p*-value**
Age (year)	70.9 ± 7.8	69.6 ± 7.1	0.598
**Sex (%)**			
Female	9 (60%)	11 (61.1%)	0.948
Male	6 (40%)	7 (38.9%)	
BMI (kg/m^2^)	29.6 ± 3.9	28.5 ± 4.2	0.448
Duration of symptoms (day)	34.9 ± 15.7	32.6 ± 15.4	0.674
Segments (L2/3/L3/4/L4/5/L5/S1)	1/3/6/5	2/3/9/4	0.855
Operated levels	1.3 ± 0.5	1.2 ± 0.4	0.739
**Comorbidity**			
Hypertension	7 (46.7%)	9 (50%)	0.739
Cardiopathy	9 (60%)	13 (72.2%)	
Lung disease	11 (73.3%)	10 (55.6%)	
Follow-up (month)	16.0 ± 3.8	17.5 ± 2.8	0.202

Values are presented as mean ± standard deviation unless otherwise indicated. BMI, body mass index.

**Table 2:** Perioperative characteristics by type of procedure.

**Variables**	**UBE-ULBD (*n* = 15)**	**OLD (*n* = 18)**	***p-*value**
Operation time (min)	67.7 ± 6.3	48.2 ± 7.2	<0.001^***^
EBL (ml)	40.6 ± 13.6	97.2 ± 19.8	<0.001^***^
Hospital stay (day)	3.5 ± 1.2	5.4 ± 1.3	<0.001^***^
**Creatine kinase (U/L)**			
Preop	61.1 ± 5.7	59.6 ± 5.3	0.455
First post-operative day	101.7 ± 15.5	330.8 ± 28.1	<0.001^***^
Seventh postoperative day	62.7 ± 5.5	63.9 ± 5.1	0.536
Perioperative complications, *n* (%)	1	5	0.117
Dural sac tearing	1	1	
Incision infection	0	4	

Values are presented as mean ± standard deviation. EBL, estimated blood loss. ^***^p < 0.001.

### Radiological results

The CSA of the paravertebral muscles in the UBE-ULBD group was significantly greater than that in the OLD group at 1 year, with a significantly lower degree of atrophy of the paraspinal muscles in the UBE-ULBD group than in the OLD group (4.81 ± 1.94 vs. 12.15 ± 6.99, *p* < 0.001, Table 3). The dural sac CSA significantly increased postoperatively in both groups, confirming that they benefited from a comparable decompressive effect (UBE-ULBD preoperative: 0.86 ± 0.09 cm^2^ vs. postoperative: 1.51 ± 0.13 cm^2^, OLD preoperative: 0.89 ± 0.10 cm^2^ vs. postoperative: 1.56 ± 0.07 cm^2^, *p* < 0.001, Table 3). The creatine kinase significantly increased in both cohorts and peaked 1 day after surgery. However, significantly lower levels were found in the UBE-ULBD group than in the OLD group (101.7 ± 15.5 vs. 330.8 ± 28.1 U/L, *p* < 0.001, Table 2). However, no significant differences were observed between the two groups preoperatively or on the seventh postoperative day.

**Table 3:** Radiographic outcome by type of procedure at the 1-year follow-up.

**Variables**	**UBE-ULBD (*n =* 15)**	**OLD (*n =* 18)**	***p*-value**
**Dural sac CSA, cm** ^2^			
Preop	0.86 ± 0.09	0.89 ± 0.10	0.428
1yr postop	1.51 ± 0.13^***^	1.56 ± 0.07^***^	0.203
Improvement percentage of dural sac CSA (%)	76.5 ± 20.0	77.3 ± 21.2	0.915
**CSA of the paravertebral muscles (PM, cm** ^2^ **)**			
Preop	30.50 ± 3.02	30.89 ± 2.89	0.685
1 yr postop	29.00 ± 2.90^*^	27.00 ± 1.81^*^	<0.022^*^
The degree of atrophy of the PM(%)	4.81 ± 1.94	12.15 ± 6.99	<0.001^***^

Values are presented as mean ± standard deviation. CSA, cross-sectional area; PM, paravertebral muscle. ^*^p < 0.05, ^***^p < 0.001.

### Clinical results

The clinical baseline parameters were similar in both groups. There were no significant differences in NRS, ODI, RMDQ, or health-related quality of life when patients were selected for surgery (*p* > 0.05, Table 4).

**Table 4:** Outcome parameters.

**Scoring system**	**UBE-ULBD (*n =* 15)**	**OLD (*n =* 18)**	***p*-value**
**NRS leg**			
Preop (mean score)	5.87 ± 1.81	6.11 ± 1.68	0.690
Postop (1 wk)	2.53 ± 0.92^***^	2.67 ± 1.02^***^	0.700
Follow-up at 1 year	2.00 ± 0.65^***^	2.06 ± 0.54^***^	0.791
**NRS lumbar**			
Preop (mean score)	6.67 ± 1.23	6.06 ± 1.51	0.219
Postop (1 wk)	3.00 ± 0.65^***^	4.00 ± 0.84^***^	<0.001^***^
Follow-up at 1 year	2.33 ± 0.72^***^	2.61 ± 0.50^***^	0.204
**ODI**			
Preop (mean score)	60.67 ± 3.50	61.22 ± 2.71	0.611
Postop (1 wk)	37.27 ± 2.25^***^	39.44 ± 2.55^***^	0.015^*^
Follow-up at 1 year	28.40 ± 3.66^***^	26.9 ± 2.82^***^	0.206
**RMDQ**			
Preop (mean score)	13.87 ± 3.50	15.39 ± 3.22	0.203
Follow-up at 1 year	5.40 ± 3.78^***^	6.67 ± 3.56^***^	0.330
**RMDQ PCS**			
Preop (mean score)	24.93 ± 5.16	24.28 ± 4.35	0.695
Follow-up at 1 year	34.73 ± 4.85^***^	34.11 ± 4.21^***^	0.696
**SF-36 MCS**			
Preop (mean score)	40.53 ± 7.13	38.67 ± 6.92	0.452
Follow-up at 1 year	50.47 ± 6.93^*^	49.67 ± 6.78^*^	0.741
**Odom's criteria (n)**			
Follow-up at 1 year	80% Satisfaction	77.8% Satisfacti	0.778
	8 Excellent	11 Excellent	
	4 Good	3 Good	
	2 Fair	1 Fair	
	1 Poor	3 Poor	

Values are presented as mean ± standard deviation. NRS, Numeric Pain Scale; ODI, Oswestry Disability Index; RMDQ, Roland and Morris Disability Questionnaire; SF-36, Short Form-36 Health Survey. ^*^p < 0.05, ^***^p < 0.001 compared to preoperative scores.

**Figure 2:**
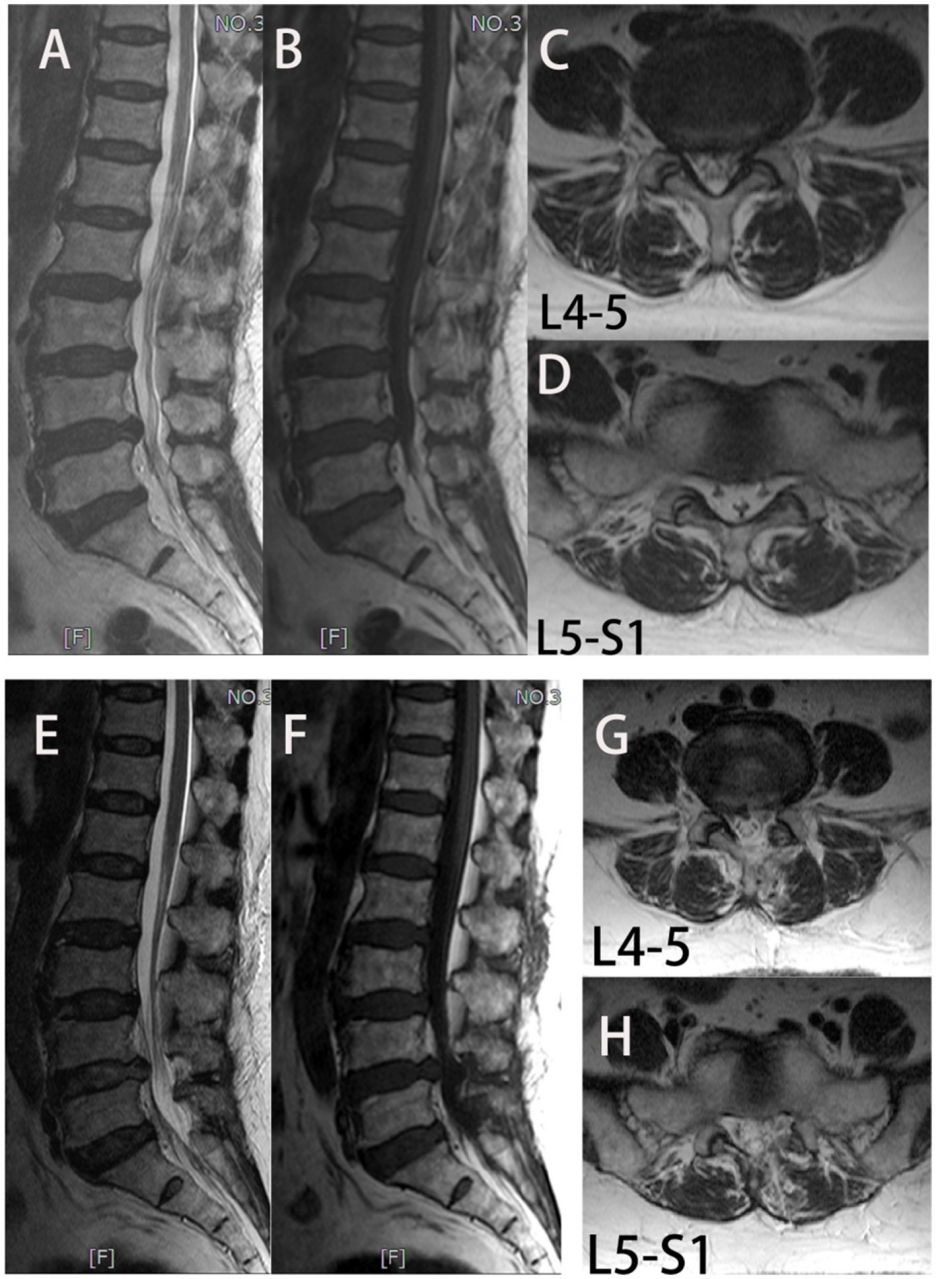
In group UBE-ULBD, a 56-year-old woman suffers from low lumbar pain accompanied by lower limb pain and numbness for more than 1 year, diagnosed to be L4-5 and L5-S1 LEL. **(A–D)** Preoperative MRI examinations showed severe LEL at L4-5 and L5-S1 levels. The patient received UBE-ULBD, and symptoms were significantly relieved after the surgery. **(F–H)** Postoperative MRI indicated completed decompression was achieved at L4-5 and L5-S1.

The NRS and ODI scores of the two groups at 1 week and 1 year after surgery were significantly improved compared with those before surgery (*p* < 0.05). However, the NRS and ODI scores of the UBE-ULBD group were significantly higher than those of the OLD group on the 7^th^ day after the operation (NRS: 3.00 ± 0.65 vs. 4.00 ± 0.84; ODI: 37.27 ± 2.25 vs. 39.44 ± 2.55, *p* < 0.01, Table 4). The RMDQ and SF-36 (physical and mental component) scores of the two groups 1 year after the operation were significantly improved compared with those before the operation (*p* < 0.05). The patients presented with clearly reduced pain-associated disability. There was no significant difference in the RMDQ and SF-36 (physical and mental component) scores between the two groups at 1-year follow-up. Due to the clinical benefits, patient satisfaction in both groups was high. In the 1-year follow-up, 80% of patients in the UBE-ULBD group scored Odom's criteria with “good” or “excellent,” as 77.8% of patients in the OLD group did (Table 4). Among the four patients with adverse symptoms, one underwent lumbar fusion surgery 3 months after surgery, and three received conservative treatment for symptom control. Typical case is shown in Figure 2.

### Surgical complications

No serious complications occurred in either group, including nerve root injury, reoperation due to postoperative hematoma, or infection within a year. There were no significant differences in the perioperative complications of dural sac tear (*n* = 1) in the UBE-ULBD group in addition to dural sac tear (*n* = 1) and incision infection (*n* = 4) in the OLD group (*p* > 0.05, Table 2). The number of postoperative incision infections in the OLD group (four cases) was greater than that in the UBE-ULBD group (zero cases), which may be linked to fat liquefaction and repeated exudation of the incision in the OLD operation area. These complications disappeared within 1 month postoperatively.

## Discussion

The LEL is a normal intraspinal fat space-occupying lesion caused by pathological hyperproliferation and accumulation of epidural fat (17). Because of stimulation or compression of the adjacent nerve root by abnormal hyperplasia of adipose tissue, patients often have a series of clinical symptoms, including lumbar pain, numbness, pain, and weakness of one or both lower limbs (2, 3, 17). Mild symptoms can be alleviated by conservative treatment; however, some patients often require surgical treatment when conservative treatment is ineffective. Traditional OLD is considered an effective surgical method for treating LEL; however, this procedure has defects (18). The UBE-ULBD aims to reduce these disadvantages because it can achieve complete resection of the diseased tissue in the spinal canal and full decompression of the spinal canal, with less tissue damage (15, 19). Our preliminary clinical results of UBE-ULBD and OLD for treating LEL disclosed that (1) after one year of follow-up, UBE-ULBD and OLD can achieve the same short-term clinical efficacy for treating LEL. (2) Compared with OLD, UBE-ULBD had lower postoperative lumbar pain NRS and ODI scores at 1 week of follow-up. (3) Compared with OLD, UBE-ULBD had less blood loss, shorter hospital stays, and less paravertebral muscle injury, which is beneficial to the postoperative rehabilitation of patients. (4) In our preliminary study, none of the patients had complications linked to this technique. Both groups achieved clinical improvement at the same time, and the overall benefit time was 1 year. The satisfaction rates of the patients in the UBE-ULBD and OLD groups were 80% and 77.8%, respectively. The two groups achieved considerable short-term clinical efficacy.

As stated previously, traditional OLD has consistently been the standard lumbar decompression technique and has yielded good clinical outcomes (20). Interestingly, in the presence of LEL with lumbar spinal stenosis, compared with other open surgeries, OLD can remove the hyperplasia and abnormal adipose tissue that compresses the dural sac and nerve root under direct vision and preserves the normal anatomy of the spine to the greatest extent (21, 22). However, the OLD approach requires extensive dissection of the paravertebral muscle tissue to expose the surgical field and greater force to continuously pull the paravertebral muscle, causing ischemic injury of the paravertebral muscle and the formation of surrounding scars (23). Increasing evidence suggests that muscle atrophy after paraspinal muscle injury accelerates spinal degeneration, leading to decreased spinal stability, postoperative pain, and dysfunction (22–24). Patients with LEL are often obese and have a thick fat layer on their waists. Simultaneously, OLD surgery can achieve a much wider range of decompression than endoscopic surgery; however, the surgical wound is larger, and the risks of incision fat liquefaction, delayed wound healing, and infection can easily occur after surgery (21, 22). Our results revealed that EBL, hospital stay duration, postoperative creatine kinase level, postoperative lumbar muscle atrophy, and postoperative incision infection rate were significantly higher in the OLD group than in the UBE-ULBD group. This demonstrates that UBE-ULBD for treating LEL can reduce the disadvantages of OLD technology and postoperative risks while achieving the same clinical efficacy as OLD technology.

The UBE-ULBD technology has several advantages over the OLD technology for the surgical treatment of LEL. First, UBE-ULBD allows the surgeon to reach the surgical target area quickly and provides a well-illuminated surgical field of view and appropriate magnification. The surgery was completed under full visualization. Second, the technique avoids unnecessary dissection of the surrounding muscle tissue and preserves the facet joints and joint capsule, which provides advantages for the rapid recovery of patients, such as reducing postoperative pain and early recovery of daily activities. Our results indicated that the EBL, hospital stay duration, postoperative creatine kinase level, postoperative lowbar muscle atrophy, and postoperative incision infection rate were significantly lower in the UBE-ULBD group than in the OLD group. The short-term (1 week after surgery) lower lumbar pain NRS and ODI scores in the UBE-ULBD group were substantially better than those in the OLD group. Third, the spinal canal of LEL is filled with abnormal hyperplasia of adipose tissue, accompanied mainly by bilateral nerve root compression, and may even be accompanied by disc herniation and bone stenosis of the nerve root canal. UBE-ULBD can visually remove abnormal adipose tissue in the spinal canal and completely decompress bilateral nerve roots, making it wider and safer than the OLD technology. Fourth, UBE-ULBD has the advantages of a shorter learning curve, wider decompression range, and higher safety than uniaxial transforaminal endoscopy for LEL. Our results disclosed that the cross-sectional area of the dural sac in the two groups was significantly improved compared to that before the operation, and there was no significant difference in the improvement percentage of the dural sac CSA between the two groups. In many cases, LEL combined with bilateral nerve root canal stenosis is difficult in traditional hemilaminectomy decompression.

Conversely, total laminectomy decompression aggravates iatrogenic injury to the posterior structure of the spine (25). To the best of our knowledge, no prospective study has compared the advantages, disadvantages, and clinical efficacy of UBE-ULBD and OLD for treating patients with LEL. In this study, we emphasize that the UBE-ULBD technique is safe and successful in treating patients with LEL, and significant clinical improvement in patients with LEL persists for at least 1 year. We believe that this is the first prospective comparison of UBE-ULBD and OLD for treating patients with LEL.

The characteristics of the LEL patient cohort in this investigation were comparable to those reported in other studies. The LEL mostly occurs in the lumbosacral segment, and 32% of the patients have glucocorticoid-related LEL. More than 50% of the patients had metabolic syndrome, and LEL patients were mostly male. Overall, these data are consistent with previous research results (16, 18). Visceral fat distribution may be associated with epidural fat accumulation in the spinal canal. In this research, UBE-ULBD or OLD surgery was performed according to the individual decisions of the spine surgeon and patient's wishes. Since the LEL lesion is not limited to the level of the intervertebral disc but is located at the entire height of the vertebral body, hemilaminectomy may be necessary in some multisegmental cases. Unilateral hemilaminectomy and bilateral spinal canal decompression were performed in both groups. This technique allows decompression of the dural sac along the entire path and reduces perioperative bleeding and tissue damage.

One limitation of this study was the small number of recruited patients, which was associated with a low prevalence of symptomatic LEL. Moreover, the outcomes of patients who received the best drug treatment for LEL have not been investigated. Consequently, there is a comparison between surgical and conservative treatments of symptomatic LEL, and this comparison should be conducted in future studies. Finally, this was a single-center prospective observational study. The sample size was relatively small, and the follow-up time was short. These results require further confirmation in prospective multicenter studies.

Among the patients who underwent spinal MRI, the prevalence of spinal epidural lipomatosis was 2.5% in those with and without spinal-related symptoms (26). The prevalence of symptomatic spinal stenosis is approximately 6% (27). Correspondingly, spine-related symptoms, such as low lumbar pain and sciatica caused by LEL, are easily missed or misdiagnosed as lumbar degenerative diseases (28). Therefore, it is crucial to evaluate the application of standardized and risk-free treatment alternatives. This study demonstrated that UBE-ULBD decompression for LEL patients is a surgical method with less trauma, safety, low complication rate, and long clinical benefit time.

## Conclusion

In the current study, UBE-ULBD achieved good surgical results after decompression without complications in patients with LEL. To treat LEL patients with bilateral nerve root stenosis, UBE-ULBD overcomes the limitations associated with intervertebral foramen and microscope channel technology. Compared with open surgery, it achieved a more consistent clinical effect, less trauma to the paravertebral muscles, faster clinical recovery, and a reduced incidence of paravertebral muscle atrophy and late low lumbar pain. Accurate diagnosis based on MRI and clinical symptoms is necessary, and thorough decompression based on analysis of the pathological anatomy of the spinal and nerve root canals is essential. The UBE technology may replace traditional surgery as the standard procedure for treating LEL.

## Data availability statement

The original contributions presented in the study are included in the article/supplementary material, further inquiries can be directed to the corresponding author.

## Ethics statement

The studies involving humans were approved by Ethics Committee of Mianyang Third People's Hospital. The studies were conducted in accordance with the local legislation and institutional requirements. The participants provided their written informed consent to participate in this study.

## Author contributions

BT: Conceptualization, Formal analysis, Investigation, Methodology, Project administration, Supervision, Validation, Writing—original draft, Writing—review & editing. Y-hZ: Data curation, Formal analysis, Project administration, Resources, Writing—original draft. CL: Conceptualization, Data curation, Writing—original draft. J-yO: Data curation, Formal analysis, Software, Writing—review & editing. Y-bW: Data curation, Software, Writing—original draft. Z-hS: Writing—review & editing. Q-YY: Investigation, Supervision, Writing—review & editing.
